# Interaction of vaginal microbiota and biomarkers in Premature rupture of membranes: from bench to beside

**DOI:** 10.3389/fimmu.2025.1642942

**Published:** 2025-08-14

**Authors:** Yudi Deng, Yang Li, Tiana Liu, Fuju Wu

**Affiliations:** Department of Obstetrics and Gynecology, The Second Hospital of JilinUniversity, Changchun, Jilin, China

**Keywords:** preterm premature rupture of membranes (PROM), vaginal microecology, bacterial vaginosis (BV), inflammation, oxidative stress

## Abstract

Preterm premature rupture of membranes (PROM) is a critical obstetric complication endangering maternal and neonatal health, with growing evidence linking vaginal microecology to its pathogenesis. This review synthesizes the relationship between vaginal microbiota and PROM risk, as well as microecology-targeted prevention and management strategies. A balanced vaginal microbiome, dominated by lactobacilli that maintain an acidic protective environment, is essential for reproductive health. Dysbiosis—marked by reduced lactobacilli and increased pathogens like Gardnerella and Atopobium—impairs local immunity, weakens fetal membranes, and elevates PROM risk, with bacterial vaginosis (BV) strongly associated with this condition. Pathogenic overgrowth activates inflammatory (via TLR-mediated IL-1β, TNF-α, IL-6 overproduction) and oxidative stress pathways: pro-inflammatory cytokines promote cervical ripening, induce matrix metalloproteinases (MMPs) to degrade fetal membrane collagen, while reactive oxygen species (ROS) directly damage structural proteins, compromising membrane integrity. Monitoring inflammatory/oxidative stress biomarkers (e.g., cytokine levels, ROS activity) enables early risk assessment. Potential interventions include probiotics to restore microbial balance, antioxidants/immunomodulators to counteract stress/inflammation, and MMP inhibitors to preserve membrane structure, all aiming to improve pregnancy outcomes. In conclusion, vaginal microecology plays a pivotal role in PROM development, underscoring the need for early microecological monitoring. Future research should dissect mechanistic complexities and develop precision tools for preterm labor management.

## 1 Introduction

Premature rupture of membranes (PROM), especially when it happens prior to 37 weeks of gestation, is a common and significant pregnancy complication that impacts a considerable number of expectant mothers globally (2020) (1). The incidence of preterm premature rupture of membranes (PPROM) frequently results in early labor, which may lead to various negative perinatal consequences, such as neonatal respiratory distress syndrome, sepsis, necrotizing enterocolitis, and developmental disorders in infants (2). These conditions significantly impact neonatal survival rates and may also lead to irreversible long-term developmental damage. Moreover, PPROM increases the risk of maternal infection, particularly when membranes are ruptured, as bacteria may ascend and invade the amniotic cavity, potentially causing severe infectious complications such as chorioamnionitis (3, 4). Therefore, it is clinically crucial to conduct a thorough investigation into the etiology of PPROM and its potential effects on the health of mothers and infants to reduce the rate of preterm birth and enhance the prognosis for both mothers and infants.

The intricate and diverse mechanisms contributing to PPROM are often linked to elements like physical harm to the fetal membranes, immune system reactions, and the presence of infections. Recent research has increasingly highlighted the importance of vaginal microbiota in preterm premature rupture of membranes (PPROM) (5, 6). A balanced vaginal microbiota is defined by a dominance of *Lactobacillus* species (7) which helps sustain the vagina’s acidic conditions by generating lactic acid, thereby suppressing pathogen development. In contrast, an imbalance in the vaginal microbiota results in fewer lactobacilli and a rise in harmful bacteria (8). This imbalance can disturb the local immune response and compromise the membranes, which may elevate the risk of membrane rupture. Bacterial vaginosis (BV), commonly seen as a sign of vaginal microbial imbalance, has shown a significant correlation with an increased risk of PPROM (9). Moreover, BV is frequently associated with heightened levels of pro-inflammatory cytokines such as IL-6 and IL-8 (10), which contribute to membrane rupture by enhancing cervical ripening and stimulating uterine contractions. Furthermore, changes in certain biochemical markers are recognized as important indicators for the early identification of PPROM. In recent studies, fluctuations in the levels of these biochemical markers have been recognized as promising biomarkers for evaluating the cervicovaginal environment and predicting the risk of premature rupture of the membranes (11, 12). The objective of this paper is to conduct a comprehensive examination of the influence of vaginal microbiota and biochemical markers on PPROM. Additionally, the study will explore the potential for enhancing pregnancy outcomes through the early monitoring of these biomarkers. This research is of significant importance for elucidating the mechanisms underlying PPROM and for the development of effective intervention strategies.

## 2 Overview of vaginal microbiota

### 2.1 Normal vaginal microbiota composition

To comprehend the traits of both healthy and dysbiotic cervicovaginal microbiota, it is crucial to acknowledge the intricacies of this microbial ecosystem and its significant influence on female reproductive health. The phrase ‘vaginal microbiota’ pertains to the diverse microbial community that inhabits the lower genital tract of females. The makeup and equilibrium of this microbiota are essential for preserving reproductive health.Typically, the vaginal microbiota is highly specific and exhibits low diversity, with a predominance of certain beneficial bacterial species, particularly *Lactobacillus*. The combined activities of these microorganisms lead to the development of a stable micro-ecosystem, which is essential for preserving the health of the female reproductive system (13). In the vagina of a healthy woman, *Lactobacillus* is the predominant beneficial bacterial species, which maintains the acidic environment through the secretion of lactic acid and other metabolic products, thereby inhibiting the growth of harmful pathogens (14). *Lactobacillus* bacteria are the primary species within the vaginal microbiota, and their dominance is considered an indicator of vaginal health. Indeed, the cervicovaginal microbiota comprises at least five principal community state types (CSTs), each characterized by a dominant *Lactobacillus* species (15) (**Table 1**).

**Table 1:** Five principal community state types of cervicovaginal microbiota in healthy woman.

CST type	Dominant bacteria	Function	Clinical significance	References
CST I	*Lactobacillus crispatus*	Dominant acidifier: Sustains optimal pH ≤ 4.5 barrier against pathogens	Associated with vaginal health, low infection and preterm birth risk	(16)
CST II	*Lactobacillus gasseri*	Dominant lactic acid producer: maintains an acidic environment, supports normal pregnancy, and reduces the risk of infection.	Associated with normal pregnancy, low infection risk	(17)
CST III	*Lactobacillus iners*	Weak acidifiers:have a weaker acidifying effect, and their clinical significance is controversial.associated with variable health outcomes, including the possibility of bacterial vaginosis	Controversial: Linked with both health and BV	(16)
CST IV	*Lactobacillus jensenii*	Lactic acid-producing acidifiers: maintain acidic pH levels, associated with low infection rates and favorable pregnancy outcomes.	Associated with low infection risk, good pregnancy outcomes	(18)
CST V	*L. iners & anaerobic bacteria (Gardnerella, Prevotella)*	Associated with dysbiosis and increased pathogenic activity	Linked with vaginal dysbiosis, increased infection risk	(19)

BV, Bacterial vaginosis.

### 2.2 Other common commensal bacteria

In addition to *Lactobacillus*, the vagina is inhabited by a variety of other commensal bacteria.In a healthy state, these bacteria do not induce disease and play a role in sustaining the balance of the vaginal microecological environment to a certain degree (16). These commensal bacteria are typically found in microenvironments dominated by *Lactobacillus*. However, when there is a reduction in *Lactobacillus* numbers, these bacteria may proliferate uncontrollabl. For example, *Bifidobacterium* spp. are predominantly located within the gut, although they can also be identified in the vagina in certain instances. In BV patients, bifidobacteria can coexist alongside *Gardnerella* and anaerobic bacteria, but they cannot fulfil the core function of *lactobacilli*. They produce lactic acid and serve as a defense against potential pathogens, but the effect was not significant (20). As another illustration, *Gardnerella vaginalis* is less prevalent in individuals with optimal vaginal health; however, its abundance markedly increases in cases of BV (21). Although it is an important marker of vaginal dysbiosis, it may also be present as a normal commensal in some individuals. Additionally, an increase in the anaerobic bacteria *Prevotella* spp. and *Mobiluncus* spp. is typically observed in cases of vaginal dysbiosis, particularly in individuals diagnosed with BV. The presence of these organisms is directly contributes to an inflammatory response and an elevated vaginal pH level (15, 22).

### 2.3 Physiological functions of vaginal microbiota

#### 2.3.1 Maintain a stable vaginal microenvironment

*Lactobacillus* is essential for preserving the stability of the vaginal microenvironment. Its main physiological role involves metabolizing glycogen to generate lactic acid, which helps to sustain the vagina’s acidic environment (23). As a result, this process usually keeps the vaginal pH between 3.5 and 4.5 (24) a spectrum that has been demonstrated to effectively curb the proliferation of different pathogens. The primary functions of vaginal microbiota are to maintain the stability of the vaginal microenvironment and to defend against pathogens that invade the vagina. The metabolites produced by beneficial bacteria, such as *Lactobacillus*, along with their immunomodulatory mechanisms, enable vaginal microbiota to serve as a natural defense barrier for the female reproductive tract. The vast majority of women (35/36, 97%) who underwent vaginal delivery at term exhibited a vaginal microbiome characterised by >75% abundance of Lactobacillus spp. Furthermore, 83% (30/36) of these women demonstrated Lactobacillus spp. abundance levels exceeding 98%. Samples obtained prior to PPROM were comparatively enriched for intermediate or Lactobacillus spp. depleted communities (PPROM; 14/60, 23% vs. Control; 1/36, 3%, P Farrell = 0.011), decreased total Lactobacillus spp. abundance (PPROM; 79% vs. Control; 96%, P Farrell = 0.016) and increased richness (total number of species observed, PPROM; 65 vs. Control; 10, P Farrell = 0.0086) (25). Rupture of the amniotic membrane takes place in the middle to late stages of pregnancy (24-29 + 6 and 30-36 + 6 weeks of gestation), prompting researchers to label these two periods as the ‘immune clock.’ (25–27). Furthermore, Zheng et al. (28) and Juliana et al. (29) underscore the significance of preserving the natural equilibrium of the vaginal microbiota throughout the gestational period.

#### 2.3.2 Resistance to pathogen invasion

An additional important function of the vaginal microbiome is its protective role against pathogens, achieved via various mechanisms. During pregnancy, increased concentrations of vaginal oestradiol and glycogen lead to greater vaginal acid levels, subsequently fostering the dominance of *Lactobacillus*. This genus is able to directly prevent the proliferation of harmful bacteria by influencing pH levels and producing antimicrobial compounds. Additionally, it enhances the host’s immune response, thereby strengthening the body’s defenses (30). The four principal mechanisms by which *Lactobacillus* resists pathogen invasion are as follows: the phenomenon of competitive exclusion, defined as *Lactobacilli* competing for adhesion sites by preferentially occupying receptor sites on vaginal epithelial cells, thus preventing the colonization of these cells by pathogens (31). The process of immunomodulation refers to the alteration of the immune system’s response to external stimuli. *Lactobacillus* has the ability to stimulate the release of anti-inflammatory cytokines, including IL-10, and simultaneously reduces the overproduction of pro-inflammatory components through its interactions with vaginal epithelial cells, which helps to deter undesirable inflammatory responses (32, 33). Furthermore, *Lactobacilli* have been shown to enhance local immune defenses by stimulating the mucosal immune system (MALT) (34). The secretion of antimicrobial substances is a key defense mechanism employed by these microorganisms. In addition to lactic acid and hydrogen peroxide, *Lactobacillus* lactis is capable of secreting bacteriocins and other antimicrobial peptides. Antimicrobial agents possess the ability to directly suppress the proliferation of harmful bacteria or eliminate them by compromising their cell walls and membrane structures (35). Various antimicrobial substances linked to the protection of vaginal epithelial cells, such as neutrophil gelatinase-associated lipocalin, calcium-binding proteins, and hyaluronic acid, are selectively stimulated by *Lactobacillus casei L. iners* (36). The symbiotic relationship between the host and microorganisms is sustained; the vaginal microbiota significantly contribute to maintaining the integrity of the mucosal barrier against external pathogens through their interaction with the host immune system. A dysbiotic vaginal microbiota facilitates the penetration of pathogens through the mucosal barrier, thereby precipitating infection and inflammation (37).

## 3 Biochemical markers associated with PROM of the fetal membranes

Recent studies have extensively examined the importance of biochemical markers in forecasting premature rupture of the membranes. Research indicates a strong connection between particular inflammatory factors, elements related to oxidative stress, and markers like matrix metalloproteinases with both the structural integrity of the membranes and the inflammatory response (38). The subsequent section will examine the particular roles of inflammation, oxidative stress, and additional biochemical markers in the context of premature rupture of the membranes.

### 3.1 Inflammation-related biochemical markers

The integrity of the fetal membrane relies on both its mechanical strength and the regulation of local and systemic inflammatory responses (39). A substantial body of evidence from numerous studies suggests that the inflammatory response plays a crucial role in the pathogenesis of premature rupture of membranes, particularly concerning the involvement of various inflammatory cytokines and mediators (40–42). A case-control study of patients in the FTB, PTB, PROM and pPROM groups (n > 6) revealed that: TNF-α, IL-6 and ADAMTS9 mRNA levels were significantly higher in the PROM and pPROM groups (p < 0.001). (42). Furthermore, evidence indicates that a reduction in *Lactobacillus* and an increase in pathogenic bacteria (e.g., *Sneathia* spp.) are associated with increased fragility of the fetal membranes and the onset of early neonatal sepsis (EONS) (42).

#### 3.1.1 Various inflammatory cytokines

Research has shown that the microbiota associated with premature rupture of membranes, including *Gardnerella vaginalis* and Atopobium vaginae, are significantly linked to increased pro-inflammatory factors (40, 43–45). In particular, pro-inflammatory factors such as IL-1 and IL-6, which are markedly elevated in the inflammatory vaginal environment, exacerbate cervical ripening and fetal membrane fragility, thereby heightening the risk of PROM (46) (**Table 2**).

**Table 2:** Inflammatory markers and PROM risk.

Inflammatory marker	Mechanism of action	Impact on PROM risk	References
IL-1	Key initiator of acute inflammatory response. Elevated during PROM, activates NF-κB signaling.	Increases inflammation, weakens membranes, raises PROM risk.	(43, 44, 46)
IL-6	Pro-inflammatory cytokine secreted by monocytes, macrophages, and fetal membrane cells.	Elevated in PPROM patients, early PROM marker, increases rupture risk.	(42, 47–49)
IL-1β	Regulates PI3K/AKT pathway, induces IL-6 production, activates NLRP3 inflammasome.	Increases cytokines, disrupts ECM, heightens PROM risk.	(41, 45, 50–53)
TNF-α	Produced by macrophages and T cells, key in infectious inflammation.	Promotes MMP expression, degrades ECM, increases membrane rupture risk.	(42, 54, 55)

IL-1, Interleukin 1. PROM, Preterm Rupture of Membranes. NF-κB, Nuclear Factor kappa-light-chain-enhancer of activated B cells.

IL-6, Interleukin 6; PPROM, Preterm Prerupture of Membranes; IL-1β, Interleukin 1 Beta; PI3K, Phosphoinositol-3 Kinase; AKT, Protein Kinase B; NLRP3, NOD-like Receptor Family Pyrin Domain Containing 3. ECM, Extracellular Matrix; TNF-α, Tumor Necrosis Factor Alpha; MMP, Matrix Metalloproteinase.

#### 3.1.2 Mediator of inflammation

Prostaglandins (PGs) are biologically active lipid molecules metabolized from arachidonic acid found in cell membranes. They are essential in the body’s inflammatory response and in facilitating uterine contraction (56). Prostaglandin E2 (PGE2) and prostaglandin F2α (PGF2α) are two prostaglandins closely associated with pregnancy and childbirth; additionally, they might play a role in the premature rupture of membranes.PGE2 is particularly significant in the onset of labor, primarily by regulating cervical softening and initiating uterine contractions (57). Research indicates that in instances of preterm premature rupture of membranes (PPROM), prostaglandin E2 (PGE2) exacerbates the inflammatory response by activating local immune cells and promoting the secretion of interleukin-1 (IL-1) and interleukin-6 (IL-6) (58). Concurrently, prostaglandin F2α (PGF2α) is essential in facilitating labor progression by inducing contractions of the uterine muscles. Importantly, the amniotic fluid from patients experiencing PPROM demonstrates significantly increased levels of PGF2α, highlighting its crucial function in the contractions occurring after membrane rupture (57, 59, 60).

### 3.2 Oxidative stress-related biochemical markers

**Figure 1:**
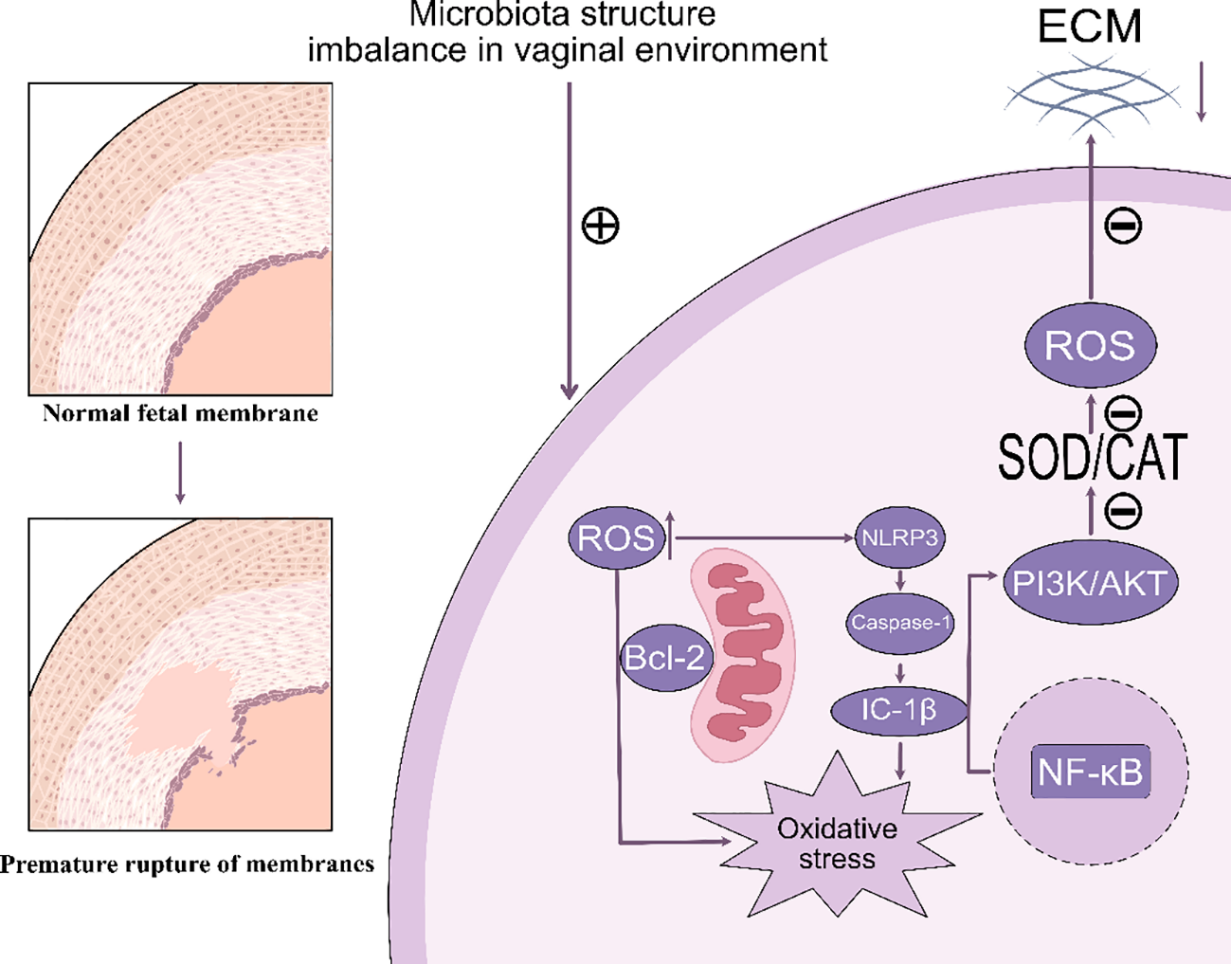
This image illustrates how microbial structural imbalances within the vaginal environment contribute to the transition from normal fetal membranes to premature rupture. The microbial imbalance generates reactive oxygen species (ROS), which activate NLRP3 inflammatory vesicles and promote the maturation of IL-1β via caspase-1. This process subsequently activates NF-κB and increases oxidative stress. Concurrently, ROS influence the PI3K/AKT and SOD/CAT pathways, resulting in damage to the extracellular matrix (ECM), which can ultimately lead to premature membrane rupture. ECM, Extracellular Matrix. ROS, Reactive Oxygen Species. SOD, Superoxide Dismutase. CAT, Catalase. PI3K, Phosphoinositol-3 Kinase. AKT, Protein Kinase B. NF-κB, Nuclear Factor kappa-light-chain-enhancer of activated B cells. NLRP3, NOD-like Receptor Family Pyrin Domain Containing 3. Bcl-2, B-cell CLL/lymphoma 2. Caspase-1, Cysteinyl aspartate specific proteinase 1, IC-1β, Interleukin 1β.

Abbreviation: ECM, Extracellular Matrix. ROS, Reactive Oxygen Species. SOD, Superoxide Dismutase. CAT, Catalase. PI3K, Phosphoinositol-3 Kinase. AKT, Protein Kinase B. NF-κB, Nuclear Factor kappa-light-chain-enhancer of activated B cells. NLRP3, NOD-like Receptor Family Pyrin Domain Containing 3. Bcl-2, B-cell CLL/lymphoma 2. Caspase-1, Cysteinyl aspartate specific proteinase 1, IC-1β, Interleukin 1β.

Oxidative stress refers to a physiological state that occurs due to an overproduction of reactive oxygen species or inadequate performance of the antioxidant defense mechanisms during the body’s metabolic activities (61). This condition has been identified as a major contributing element to the onset of preterm premature rupture of membranes, particularly in instances of preterm delivery (45, 62). This condition increases the vulnerability of the fetal membranes by disrupting their cellular structure and modulating the inflammatory response (63) (**Figure 1**).

#### 3.2.1 Reactive oxygen species and antioxidant enzymes

ROS are naturally occurring by-products of cellular metabolism, including superoxide, hydrogen peroxide, and hydroxyl radicals (64). Under standard conditions, the human body has an antioxidant defense mechanism that can eliminate these reactive oxygen species. Nonetheless, the existence of a microbial community largely made up of *non-Lactobacillus* sp*ecies* might increase ROS production, thereby worsening cellular and tissue harm (65). An excessive accumulation of ROS within the fetal membrane can lead to embrittlement and eventual rupture of the membrane structure. Antioxidant enzymes, such as superoxide dismutase (SOD) and catalase (CAT), play a pivotal role in the scavenging of ROS. SOD converts superoxide radicals into hydrogen peroxide, which is then broken down into water and oxygen by CAT. These antioxidant enzymes are essential for maintaining cellular redox homeostasis through the regulation of ROS production and scavenging (66). In patients with PPROM, it has been demonstrated that the IL-1β-induced PI3K/AKT pathway can facilitate ROS generation, while the activity of antioxidant enzymes is significantly diminished. This evidence suggests that oxidative stress may be a critical mechanism underlying the fragility and rupture of the fetal membrane (67).

#### 3.2.2 Oxidative stress

Reactive oxygen species (ROS) have the capability to directly damage collagen and elastin, which serve as structural proteins within the extracellular matrix (68). Collagen in the fetal membranes is essential for maintaining their strength and elasticity. ROS-mediated oxidative damage to collagen fibers results in their weakening, which subsequently increases the likelihood of membrane rupture (69). Concurrently, this process triggers the NLRP3 inflammatory vesicle, amplifying the inflammatory response and cytokine secretion within the fetal membranes (45, 51, 53). Such reactions further compromise the integrity of collagen fibers in the fetal membranes, thereby increasing their fragility. An excess of ROS may also activate apoptotic pathways by damaging mitochondrial membranes. The accumulation of apoptotic cells within fetal membrane tissues can weaken the structural integrity of the membrane, thereby elevating the risk of PPROM (70).

### 3.3 Other relevant biochemical markers

In addition to biochemical markers associated with inflammation and oxidative stress, matrix metalloproteinases (MMPs) and their inhibitory factors (TIMPs), among others, have been identified as playing a significant role in premature rupture of the membranes.

#### 3.3.1 Matrix metalloproteinases and their inhibitors

MMPs are a class of enzymes that degrade extracellular matrix proteins and play a crucial role in tissue remodeling and the renewal of the extracellular matrix (71). However, excessive activation of MMPs can disrupt the extracellular matrix, leading to an increased risk of premature rupture of membranes. MMP-9 is a particularly abundant matrix metalloproteinase found in fetal membrane tissue and is primarily responsible for the degradation of collagen and elastin. In patients with PPROM, there is a significant increase in MMP-9 activity, resulting in the degradation of collagen fibers and a reduction in membrane strength (72).

Tissue inhibitor of metalloproteinases (TIMP-1) serves as an endogenous inhibitor of MMPs preventing their excessive degradation of the extracellular matrix through binding interactions (71). A reduction in TIMP-1 activity may result in uncontrolled MMP activity, thereby exacerbating membrane degradation in the context of premature rupture of the membrane (73).

#### 3.3.2 Fibronectin

Fibronectin, a multifunctional protein found within the extracellular matrix, is crucial for cell adhesion, migration, and tissue repair.The findings showed a notable rise in fibronectin concentrations in cervicovaginal secretions from patients who were undergoing premature rupture of the membranes. This finding implies that fibronectin may act as an early biochemical marker for membrane rupture (74). Evidence suggests that fibronectin is a crucial factor in preserving the structural integrity of fetal membranes due to its role in extracellular matrix remodeling and the fetal membrane repair processes (55, 75).

## 4 Direct interaction of vaginal microbiota with biochemical markers

The microbiota of the vagina is crucial for ensuring the health of the female reproductive system, especially in thwarting infections and maintaining the ecological balance within the vaginal environment. However, a dysbiotic microbiota—characterized by a reduction in *Lactobacillus* and an increase in pathogenic bacteria—can trigger a range of adverse biological responses that influence the expression levels of biochemical markers. These biochemical markers not only serve as diagnostic indicators of inflammatory and oxidative stress states but also interact with changes in vaginal microbiota, creating positive or negative feedback mechanisms that further impact reproductive health. This paragraph will examine the direct interactions between vaginal microbiota and biochemical markers, with a particular emphasis on the effects of dysbiosis on biochemical markers and the counteracting influences of these markers on the balance of vaginal microbiota.

### 4.1 Influence of vaginal microbiota on biochemical markers

The stabilisation of the vaginal microbiota is largely dependent on the presence of *Lactobacillus*, with strains such as *Lactobacillus crispatus* and *Lactobacillus gasseri* playing a particularly significant role.The lactic acid produced by these bacteria maintains the acidic environment of the vagina, which inhibits the multiplication of pathogenic bacteria.In the event of a dysbiotic vaginal microbiota, as observed in cases of bacterial vaginosis or anaerobic bacteria, a localised pro-inflammatory response is initiated, accompanied by a notable elevation in the levels of inflammatory biochemical markers, including IL-1, IL-6 and TNF-α.

#### 4.1.1 Mechanisms of elevated inflammation-related biochemical markers due to dysbiosis

**Figure 2:**
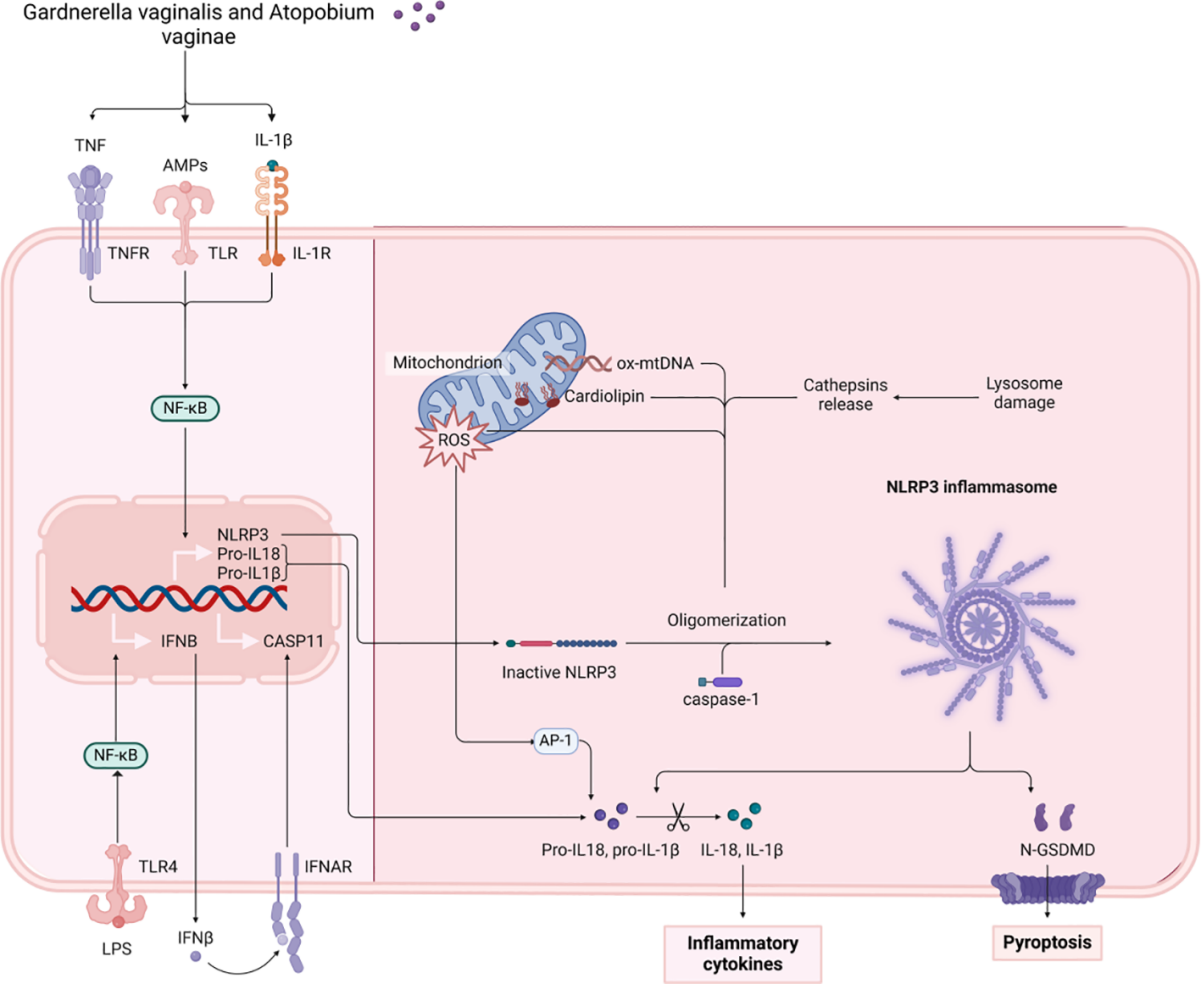
The figure illustrates the mechanisms by which *Gardnerella vaginalis* and *Atopobium vaginae* activate NLRP3 inflammatory vesicles through TNF, LPS, and other pathways. This activation results in the release of the inflammatory factors IL-1β and IL-18, subsequently triggering cellular pyroptosis. TNF, Tumor Necrosis Factor. NF-κB, Nuclear Factor kappa-light-chain-enhancer of activated B cells. LPS, Lipopolysaccharide. TNFR, Tumor Necrosis Factor Receptor. TLR, Toll-like Receptor. IFNβ, Interferon Beta. AMPs, Antimicrobial Peptides. IL-1β, Interleukin 1 Beta. CASP11, Caspase 11. IL-1R, Interleukin 1 Receptor. IFNAR, Interferon Alpha and Beta Receptor. ROS, Reactive Oxygen Species. NLRP3, NOD-like Receptor Family Pyrin Domain Containing 3. CARD, Caspase Recruitment Domain. ox-mtDNA, Oxidized Mitochondrial DNA. Cathepsins, Proteolytic enzymes found in lysosomes. N-GSDMD, N-terminal Gasdermin D. API, Apoptosis Inhibitor. IL-18, Interleukin 18.

Antimicrobial peptides (AMPs) play a crucial role in maintaining the physiological barrier of the vagina. They contribute to microbiota stability by inhibiting pathogenic organisms (76). Human beta-defensin-2 (HBD-2) is a vital antimicrobial peptide that protects against microbial invasion through the innate immune system (77). Various research works indicate that levels of HBD-2 are markedly lower in individuals suffering from bacterial vaginosis (BV) while being heightened in healthy pregnant women (78). A decrease in HBD-2 levels has been observed in instances of dysbiotic vaginal microbiota, characterized by an imbalance in microbiota dominated by pathogenic bacteria such as *Gardnerella vaginalis*. Such imbalances indicate a disruption of vaginal microecology (79, 80). Moreover, reduced concentrations of HBD-2 have been demonstrated to hinder immune function and elevate the likelihood of premature rupture of membranes (PROM) as a result of an inflammatory reaction that raises pro-inflammatory biochemical indicators, such as IL-6 and TNF-α (81). Concurrently, the proliferation of pathogenic bacteria, such as *Gardnerella vaginalis* and Atopobium vaginae, which are indicative of dysbiotic vaginal microbiota, leads to the destruction of epithelial cell integrity and stimulates the release of pro-inflammatory factors by local immune cells (13, 82). Pro-inflammatory factors have been shown to activate inflammatory signals via the NF-κB and AP-1 pathways, thereby amplifying local inflammatory responses. For example, IL-1β increases the release of IL-6 by activating the PI3K/AKT signaling pathway via the NLRP3 inflammasome, which facilitates the maturation of IL-1β through the activation of caspase-1 (83). As pro-inflammatory factor levels rise, there is a substantial influx of immune cells from local tissues, further disrupting the vaginal environment. The persistence of this inflammatory response leads to the disruption and increased fragility of the fetal membrane structure, consequently heightening the risk of premature rupture of the membranes. Additionally, in women experiencing premature rupture of the membranes, specific inflammatory factors such as CXCL10, CCL26, CCL22, and IL-16 are strongly associated with CST type IV vaginal microbiota, particularly pathogenic bacteria like *Sneathia sanguinegens* (83). The concentration of these pro-inflammatory mediators tends to rise as the non-*Lactobacillus*-dominated microbiota expands. This imbalance in gut microbiota initiates a localized inflammatory reaction, leading to heightened biochemical indicators linked to inflammation and a greater likelihood of premature rupture of the membranes (**Figure 2**).

#### 4.1.2 Modulation of markers associated with oxidative stress

It has been demonstrated that the prevalence of *L. iners* fluctuates considerably during the early stages of pregnancy, with a notable increase observed as vaginal cleanliness declines (84). This indicates that the rise in biochemical markers linked to vaginal microbiota dysbiosis and oxidative stress may be attributable to the prevalence of *L. iners*, which facilitates the proliferation of pathogenic bacteria, resulting in elevated levels of ROS production. This, in turn, further exacerbates the imbalance in the vaginal environment and tissue damage (85, 86). In a usual situation, Lactobacilli hinder the growth of harmful bacteria by preserving an acidic environment and generating hydrogen peroxide (H_2_O_2_). When dysbiosis occurs, there is a decrease in HBD-2 levels, resulting in a rise of pathogenic bacteria in the vaginal area.The production of toxins and the induction of reactive oxygen species (ROS) by these bacteria lead to oxidative stress (87). ROS production results in damage and increased fragility of the fetal membrane structure, due to the attack on collagen fibres and other extracellular matrix proteins that occur within the fetal membrane (88). Concurrently, the activity of antioxidant enzymes (e.g. SOD, CAT) within the vaginal environment is diminished, thereby further compromising the capacity to avert oxidative stress (89). The available literature indicates a negative correlation between specific microbial types (e.g., *Gardnerella vaginalis* and *Atopobium vaginae*) and CXCL10 levels. This suggests that these pathogens may contribute to the disruption of vaginal microecological stability by triggering inflammation and oxidative stress (90).

Oxidative stress linked to dysbiosis increases the risk of fetal membrane rupture. Besides directly harming cellular structures, an overabundance of reactive oxygen species (ROS) further encourages the breakdown of the extracellular matrix through the activation of MMPs (90). It is evident that the inflammatory response and oxidative stress induced by dysbiosis not only increase the levels of pro-inflammatory cytokines but also enhance the expression of biochemical markers related to oxidative stress, thereby further accelerating the degradation of fetal membranes.

### 4.2 Reverse effects of biochemical markers on vaginal microbiota

#### 4.2.1 Changes in the composition of the vaginal microbiota by the inflammatory environment

In non-pregnant women, the vaginal microbiota composition is less stable, marked by a higher percentage of pathogens. In contrast, a healthy pregnancy is associated with increased stability of the vaginal microbiota, showcasing a higher prevalence of beneficial bacteria like *Lactobacillus*. This shift may serve to address the protective needs of both the fetus and the mother (91). Notably, a microbiota dominated by a single strain of *Lactobacillus iners* during the early stages of pregnancy is significantly associated with the occurrence of preterm labor (6). The research revealed that L. iners was detected in merely 85% of women who underwent preterm deliveries, while only 16% of those with full-term pregnancies showed the presence of this strain (92). Furthermore, the pro-inflammatory cytokines IL-1β and TNF-α not only promote localized inflammation but also have a considerable impact on the makeup of the vaginal microbiota (93). As levels of inflammation increase, the quantity of *Lactobacillus* bacteria diminishes, whereas the presence of anaerobic pathogens, such as *Gardnerella vaginalis* and *Atopobium vaginae*, rises. This change exacerbates dysbiosis, thereby heightening the risk of premature rupture of membranes. In women of African descent, inflammatory conditions are more conducive to the colonization of anaerobic bacteria like *Gardnerella* and *Prevotella*, resulting in a transition of the vaginal microbiota from a state dominated by *Lactobacillus* to one defined by anaerobic dysbiosis (94). A strong correlation has been observed between vaginal cleanliness, leukocyte esterase levels, and the composition of the vaginal microbiota. Specifically, the presence of leukocyte esterase correlates with a notable increase in the abundance of *L. iners*, while the abundance of L. crispatus shows a significant decline (85). The *CST IV* microbiota, characterized by the dominance of *non-Lactobacillus bacteria* in the vagina during inflammatory states, has been associated with elevated levels of several pro-inflammatory biochemical markers (95). Pro-inflammatory agents, including IL-1β, not only enhance the synthesis of HBD2 within an inflammatory context but also modify the vaginal microbiota’s composition, suppressing the growth of beneficial bacteria such as *Lactobacillus* while encouraging the increase of pathogenic bacterial populations. A study involving 317 patients diagnosed with bacterial vaginosis (BV) revealed that vaginal hBD-2 levels were 54.48% lower compared to those in the healthy control group (p < 0.01). Furthermore, a significant negative correlation was observed between Nugent scores and both hBD-1 (Spearman’s rho = -0.2118; p = 0.0001) and hBD-2 (Spearman’s rho = -0.2117; p = 0.0001) levels (78). It has been proposed that during the inflammatory process, the concentration of intravaginal AMPs, such as HBD2, increases, effectively inhibiting the reproduction of pathogenic bacteria and protecting beneficial microbiota (80, 90). This counterproductive mechanism suggests that biochemical markers play a crucial role in maintaining the equilibrium of vaginal microbiota, thereby facilitating the prevention of infection.

#### 4.2.2 Adaptive changes in bacterial microbiota under oxidative stress conditions

In the context of oxidative stress, AMPs influence the composition of the vaginal microbiota in two principal ways: first, by directly inhibiting pathogenic bacteria, and second, by modulating the local inflammatory response (96). For instance, the overproduction of ROS and other oxidative stress-related markers can adversely affect the vaginal microbiota. ROS are not only toxic to host cells but also impact the environmental conditions necessary for microorganisms to survive and thrive (97). In the presence of oxidative stress, pathogenic bacteria capable of tolerating oxidative damage, such as *Sneathia* spp., tend to dominate the vaginal environment, further exacerbating the inflammatory response and tissue damage. The survival of *Lactobacillus* in this oxidative stress environment is compromised, leading to a reduction in its population. Consequently, this reduction results in an increase in vaginal pH, creating a more favorable environment for the colonization and proliferation of pathogenic bacteria. Oxidative stress promotes the adaptive growth of pathogenic bacteria by altering the vaginal microecology, thereby heightening the likelihood of an inflammatory response and membrane rupture. This feedback mechanism suggests that elevated biochemical markers are not merely a consequence of changes in the vaginal milieu; rather, they actively contribute to the perpetuation of microbiota dysbiosis, establishing a vicious cycle (98). For example, in the context of oxidative stress, *L. iners* exhibits enhanced resilience and the ability to persist in a dysbiotic vaginal environment (85). However, the presence of *L. iners* does not effectively inhibit the colonization of other harmful bacteria and may instead serve as a marker of vaginal microbiota dysbiosis (84).

## 5 Mechanism of synergy between vaginal microbiota and biochemical markers in PROM

Recent studies have demonstrated that the vaginal microbiota and biochemical markers exert a synergistic effect on the pathogenesis of premature rupture of membranes. This paragraph will provide a detailed discussion of the mechanisms underlying the synergy between vaginal microbiota and biochemical markers in the context of PROM.

### 5.1 Synergistic effects through inflammatory pathways

#### 5.1.1 Vaginal microbiota triggers an inflammatory response and activates the expression of relevant biochemical markers

Changes in the vaginal microbiota have been demonstrated to play a pivotal role in the initiation of the inflammatory response. The usual vaginal microbiome is defined by a dominance of *Lactobacillus* species, which produce lactic acid to create an acidic setting, thereby effectively preventing the proliferation of harmful bacteria (23, 24). However, in the event of a dysbiotic vaginal microbiota, the proliferation of pathogenic bacteria, including *Gardnerella vaginalis*, *Prevotella* and *Atopobium vaginae*, is observed (99). The colonisation of pathogenic bacteria has been demonstrated to have a dual impact on the vaginal barrier function, leading to its destruction and the subsequent activation of the host’s innate immune system. This occurs through direct interaction with host cells, which in turn triggers an inflammatory response (42, 82, 100). These pathogenic bacteria have been demonstrated to activate host immune cells via the TLR (Toll-like receptor) pathway, which results in the excessive release of pro-inflammatory cytokines (e.g. IL-1β, TNF-α, IL-6) (101, 102). The TLR2/TLR1 heterodimer has been demonstrated to recognize bacterial triacylglycerol lipopeptides, such as those derived from Mycoplasma, and to activate Th17-type immune responses, which subsequently lead to pro-inflammatory reactions. The normal vaginal microbiota, particularly Lactobacillus, has been shown to suppress TLR2 expression, thereby preventing excessive inflammatory responses against commensal bacteria. However, in instances where the microbiota is disrupted, TLR2/TLR1 heterodimers activated by pathogens like Streptococcus gordonii trigger Th17 responses, resulting in inflammation and pyroptosis (103). The presence of lipopolysaccharide (LPS) has been shown to induce the activation of Toll-like receptor 4 (TLR4), thereby stimulating the nuclear factor kappa B (NF-κB) pathway and promoting the secretion of pro-inflammatory mediators such as interleukin-6 (IL-6) and vascular endothelial growth factor-A (VEGF-A). During periods of microbial imbalance, the overgrowth of potentially pathogenic bacteria, such as those associated with aerobic vaginitis, can lead to increased secretion of LPS, which in turn activates the NF-κB pathway, disrupting the balance of vaginal microbiota and exacerbating inflammation (104). TLR9 has been found in the cytoplasm of cells and is capable of recognizing non-methylated CpG dinucleotides in bacterial DNA, for instance, in Staphylococcus. This recognition process is induced via the MyD88 pathway, resulting in the secretion of IFN-γ and IL-1β. During bacterial community dysregulation, the secretion of bacterial DNA through TLR9 is induced by antibiotic action (105).

**Figure 3:**
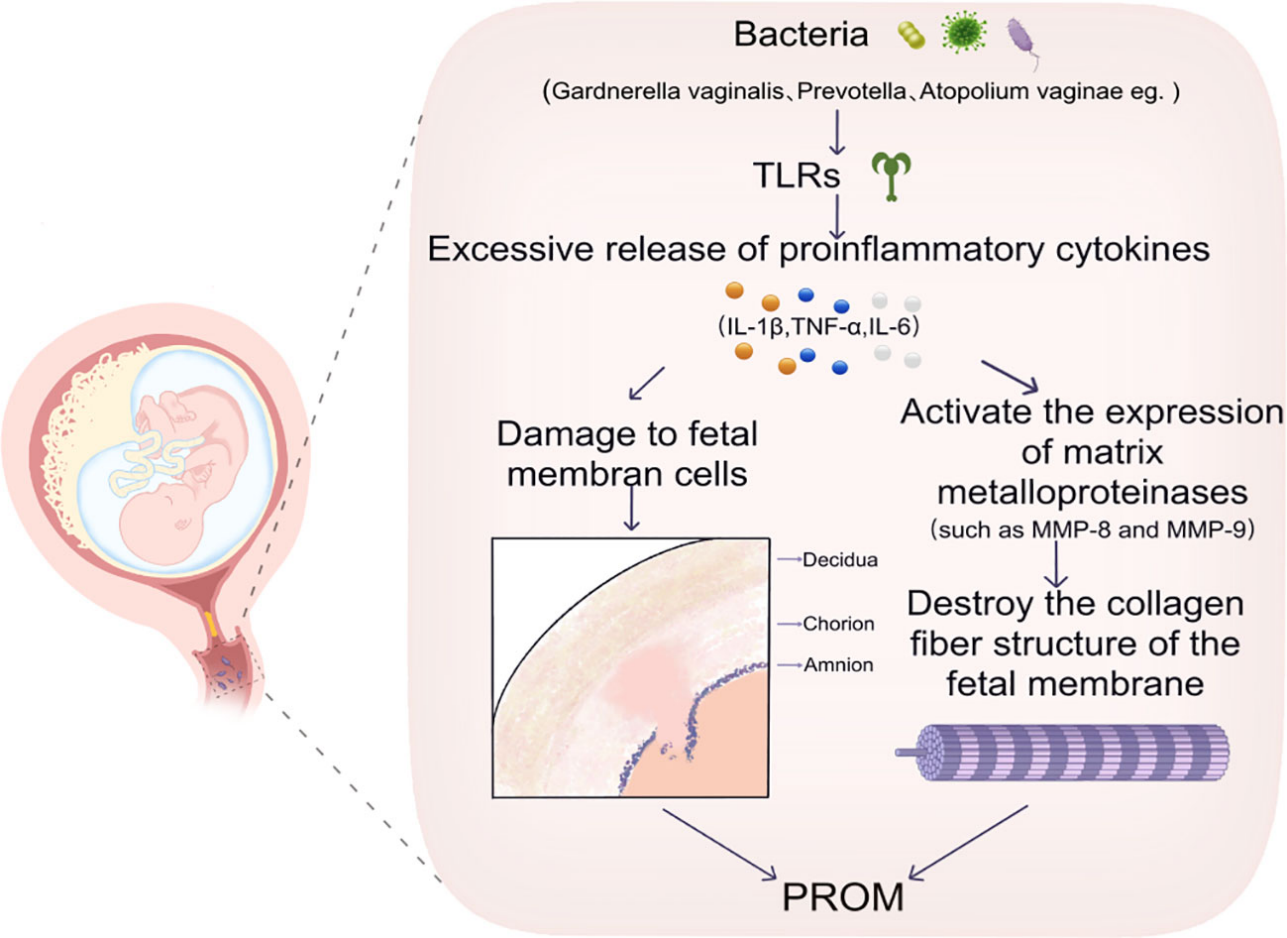
This image illustrates that certain bacteria, such as *Gardnerella vaginalis*, *Prevotella*, and *Atopobium vaginae*, induce an excessive release of inflammatory cytokines, including IL-1β, TNF-α, and IL-6, through the activation of TLRs. This activation results in damage to fetal membrane cells. Consequently, this damage triggers the expression of MMPs, specifically MMP-8 and MMP-9, which disrupts the collagen fiber structure of the fetal membranes and may ultimately contribute to PROM. TLRs, Toll-like Receptors. IL-1β,Interleukin 1 Beta. TNF-α, Tumor Necrosis Factor Alpha. IL-6,Interleukin 6.MMP-8, Matrix Metalloproteinase 8. MMP-9, Matrix Metalloproteinase 9. PROM, Preterm Rupture of Membranes.

It has been demonstrated that the levels of these inflammatory markers are markedly elevated in patients diagnosed with BV (19). The release of pro-inflammatory factors has been demonstrated to cause direct damage to fetal membrane cells (106), additionally, the expression of MMPs (e.g., MMP-8, MMP-9) is triggered (107), further disruption of the collagen fibre structure of the membranes results in an increased brittleness of the membranes (**Figure 3**).

#### 5.1.2 Biochemical markers further exacerbate inflammation leading to premature rupture of membranes

Biochemical markers in the inflammatory response serve not only as indicators of inflammation but also have the potential to directly exacerbate fetal membrane damage. Matrix metalloproteinases (MMPs) represent a category of enzymes crucial for breaking down the extracellular matrix, which is vital for the proper remodeling of fetal membranes (62, 108). However, when activated by the inflammatory response, the overexpression of MMPs leads to structural damage to the membranes. Specifically, MMP-9 and MMP-8 are released in large quantities during inflammation and infection, resulting in the degradation of crucial structural components, such as collagen fibers and elastin, thereby weakening the strength and elasticity of the membranes (71). At the same time, pro-inflammatory cytokines such as IL-1β and IL-6 worsen the damage to fetal membranes by increasing the activity of MMPs (109). Studies have demonstrated that the levels of IL-6 in amniotic fluid are significantly elevated in women experiencing premature rupture of membranes (PROM) and are strongly correlated with increased MMP-9 activity. (110). The combined effects of these pro-inflammatory factors and MMPs contribute to the premature rupture of the fetal membranes due to accelerated degradation of the extracellular matrix.

### 5.2 Synergistic effects through oxidative stress pathways

Dysbiosis of the vaginal microbiota is associated with both an inflammatory response and exacerbation of fetal membrane damage via the oxidative stress pathway (111). A reduction in *Lactobacillus* levels correlates with a significant increase in oxidative stress, which facilitates the proliferation of pathogenic bacteria such as *Gardnerella* and *Prevotella*. These bacteria produce substantial quantities of ROS, which not only directly damage fetal membrane cells but also exacerbate inflammatory responses by activating pro-inflammatory pathways (99). Elevated levels of ROS in the vagina have been significantly linked to the disruption of fetal membranes and an increased risk of preterm labor (8, 112). For instance, *Gardnerella vaginalis*, *Prevotella*, and *Sneathia* can generate considerable amounts of ROS as a result of their metabolic processes. The overproduction of ROS leads to oxidative damage to fetal membrane cells and further enhances the release of pro-inflammatory cytokines by activating inflammatory pathways such as NF-κB. Research has shown that in patients with vaginal dysbiosis, ROS levels are markedly elevated, accompanied by a reduction in the antioxidant capacity of fetal membrane cells, resulting in increased oxidative damage to these cells (113, 114). The interplay between oxidative stress and vaginal microbiota dysbiosis significantly influences the structure and function of fetal membranes, resulting in cellular-level disruptions. Reactive oxygen species (ROS) not only inflict direct damage on lipids, proteins, and DNA inside the cells of the fetal membrane but also encourage the breakdown of the extracellular matrix through the activation of matrix metalloproteinases (MMPs) (115). In patients experiencing premature rupture of membranes, elevated levels of oxidative damage markers, including lipid peroxidation products (MDA), have been detected in fetal membrane tissues, underscoring the substantial role of oxidative stress in fetal membrane injury (116). Furthermore, oxidative stress adversely affects the signaling processes of fetal membrane cells, particularly through the activation of pathways such as NF-κB and MAPK. This stimulation increases the production of pro-inflammatory elements, which, in turn, heightens the inflammatory response and the functioning of MMPs. As a result, this establishes a harmful cycle that causes the swift degradation of membranes, stemming from the synergistic impact of oxidative stress and inflammation, ultimately culminating in the premature rupture of membranes (117, 118).

### 5.3 Other synergistic mechanisms

#### 5.3.1 Co-regulation involving MMPs and others

MMPs play a crucial role in both the physiological remodeling and pathological degradation of the fetal membrane. The activation of MMPs occurs through inflammatory and oxidative stress pathways; however, their expression is also modulated by the microbiota and other metabolites (119). For instance, the vaginal microbiota, predominantly composed of *Lactobacillus* spp., not only maintains an acidic environment but also diminishes MMP activity by inhibiting the production of pro-inflammatory factors (24). Conversely, when the microbiota becomes dysbiotic, particularly with the prevalence of pathogens, MMP activity is heightened, leading to accelerated degradation of cell membranes and an increased risk of premature rupture of membranes (24, 120). Bacterial metabolites, including short-chain fatty acids (SCFAs) and lipopolysaccharides (LPS), may affect the activity of MMP through both direct and indirect pathways (121). In the context of vaginal dysbiosis, metabolites produced by pathogenic bacteria may further exacerbate fetal membrane degradation by activating MMPs (122, 123). Moreover, the degradation of fetal membranes is closely linked to the action of MMP inhibitors, such as tissue inhibitors of metalloproteinases (TIMPs). During healthy pregnancies, MMP activity is tightly regulated by TIMPs, which help maintain the structural integrity of the fetal membrane (73). However, in the presence of inflammatory and oxidative stress, TIMP expression is diminished, leading to the deregulation of MMP activity and further disruption of the fetal membrane.

#### 5.3.2 Influence on metabolism and signalling in fetal membrane cells

Vaginal microbiota and their metabolites can regulate the function and structure of fetal membranes by influencing the metabolism and signaling of fetal membrane cells (74, 124). Metabolites produced by *Lactobacillus*, including lactic acid and hydrogen peroxide, are essential for preserving the balance of fetal membrane cells and for inhibiting harmful bacteria. However, a reduction in *Lactobacillus* populations may disrupt the metabolic pathways of fetal membrane cells.

The impact of vaginal microbiota imbalance on amniotic membrane cells extends beyond mere inflammation activation; it profoundly disrupts fundamental metabolic processes and crucial signaling pathways within the cells. Dysregulation associated with bacterial vaginosis, for instance, significantly affects amino acid, carbohydrate, and energy metabolic pathways, thereby impacting the nutrient supply and energy homeostasis of amniotic membrane cells. This disruption manifests as abnormal alterations in lipid metabolic pathways, including fatty acid oxidation and membrane lipid synthesis, closely linked to insulin resistance and impaired cellular signaling (125). Concurrently, the imbalance in the microbial community directly induces excessive levels of reactive oxygen species (ROS) in amniotic fluid cells via metabolic processes, inhibiting important antioxidant enzymes such as superoxide dismutase (SOD) and glutathione peroxidase (GPX), which disrupts the balance of oxidation-reduction processes. Furthermore, this oxidative stress state activates the NLRP3 inflammasome, promoting the maturation of the IL-1β inducer, leading to collagen degradation and extracellular matrix (ECM) damage, thus increasing the risk of amniotic membrane structural damage (126). Additionally, dysbiosis has been shown to interfere with crucial non-inflammatory signaling pathways, including the reduction of antimicrobial peptides such as human beta-defensin-2 (HBD-2), weakening the innate immune barrier function, and disrupting tryptophan metabolism (e.g., kynurenine accumulation), which affects the differentiation of amniotic membrane cells and the barrier repair function through the aryl hydrocarbon receptor (AhR) pathway. While this pathway has been extensively studied in the context of intestinal microbiota, it has received comparatively minimal attention concerning vaginal microbiota (127). Notably, these effects often occur independently of the classic inflammatory response.

Furthermore, substances produced by harmful bacteria, including lipopolysaccharides (LPS), have the capacity to trigger apoptosis and inflammatory reactions in fetal membrane cells through the activation of the TLR signaling pathway (128). The activation of the TLR pathway not only enhances the release of pro-inflammatory factors but also regulates gene expression.

In response to the negative effects of dysbiosis, metabolites of lactic acid bacteria establish a crucial protective signaling network. Lactic acid not only lowers vaginal pH to create a chemical barrier but also directly enhances the barrier function of the cervical epithelium. Clinical observations indicate that elevated levels of lactic acid are significantly associated with the upregulation of tight junction proteins, such as ZO-1 (129). Short-chain fatty acids (SCFAs), including acetate and propionate, are produced by Lactobacillus. These SCFAs activate G protein-coupled receptors, such as GPR43, thereby exerting potent anti-inflammatory effects. They promote IL-10 secretion, inhibit the NF-κB pathway, and alleviate oxidative damage (130). Simultaneously, SCFAs inhibit the assembly of the NLRP3 inflammasome and the maturation of IL-1β, thus protecting the extracellular matrix (ECM) from excessive proteolytic damage (131). Perhaps most importantly, Lactobacillus colonization can reverse the abnormalities in purine degradation and membrane lipid metabolism caused by dysbiosis through metabolic reprogramming. This process restores energy metabolism homeostasis and reduces the risk of insulin resistance. Its metabolites, such as nicotinamide, enhance the efficiency of the mitochondrial respiratory chain via the NAD+ pathway, inhibit excessive activation of p38 MAPK, and effectively maintain mitochondrial function. Consequently, this alleviates cellular stress damage and comprehensively enhances the defensive and reparative capabilities of the amniotic membrane (132).

## 6 Clinical implications based on the interaction of vaginal microbiota with biochemical markers

Recent studies have demonstrated that dysregulation of vaginal microbiota is closely linked to alterations in biochemical markers, including pro-inflammatory cytokines and MMPs. As a result, the combined evaluation and handling of vaginal microbiota paired with biochemical indicators offer novel possibilities for the clinical identification and management of premature rupture of membranes and preterm labor.

### 6.1 Diagnostic

#### 6.1.1 Combined vaginal microbiota test and biochemical marker test

In clinical diagnosis, the combined assessment of vaginal microbiota and biochemical markers can enhance the early detection of premature rupture of membranes. Traditional diagnostic methods typically depend on clinical symptoms or the identification of individual pathogens; however, the dynamics of the vaginal microbiota, particularly the balance between microbiota such as *Lactobacillus crispatus* and *Gardnerella vaginalis*, are crucial for the onset of PROM (16, 133, 134). Studies have demonstrated that dysbiosis of the vaginal microbiota, characterized by a decrease in *Lactobacillus* and an increase in anaerobic bacteria, is frequently associated with elevated levels of pro-inflammatory factors (e.g., IL-6, TNF-α) and MMPs (e.g., MMP-8, MMP-9). These biochemical markers are integral to the process of premature rupture of membranes.

By integrating vaginal microbiota testing with biochemical markers, clinicians can more effectively identify patients at risk, facilitating timely interventions. For instance, in individuals diagnosed with bacterial vaginosis (BV), elevated levels of pathogenic bacteria, such as *Gardnerella vaginalis* and *Prevotella*, closely correlate with variations in inflammatory markers. These markers can serve as crucial reference points for the early diagnosis of premature rupture of membranes (135). The combination of vaginal microbiota assessment and biochemical markers not only enhances diagnostic sensitivity and specificity but also lays the groundwork for individualized treatment approaches (136). Furthermore, the detection of interactions between pro-inflammatory cytokines and vaginal fmicrobiota may prove valuable in evaluating the progression of fetal membrane rupture. Research indicates that the inflammatory response is a significant trigger for premature rupture, with elevated inflammatory markers showing a positive correlation with structural damage to the fetal membranes (137). By routinely assessing these biochemical markers alongside microbiota status, clinicians can better predict a patient’s risk of preterm rupture of membranes and implement timely interventions.

#### 6.1.2 Exploration of new diagnostic markers

In addition to traditional markers of inflammation and MMPs, scientists have recently begun to investigate new diagnostic markers, such as the detection of vaginal metabolites and indicators of oxidative stress. For instance, the generation of ROS is closely linked to oxidative damage to fetal membranes, and certain oxidative stress markers, such as malondialdehyde (MDA), can indicate the extent of structural damage to these membranes (138, 139). Furthermore, specific metabolites, such as short-chain fatty acids (SCFAs), may reflect the relationship between microbial metabolic activity and imbalances in the vaginal environment, potentially providing a diagnostic basis for the early identification of high-risk patients (130). By exploring these novel markers, future diagnostic tools are expected to be more comprehensive and accurate, enabling effective differentiation between various types of microbial diseases and their corresponding biomarker changes, thereby offering personalized diagnostic solutions for diverse patient populations.

Novel diagnostic markers currently under investigation include specific metabolites identified through metabolomic analyses, such as lactic acid, acetic acid, and lactate (140). Dynamic changes in vaginal metabolites not only reflect the balance of the microbiota but can also serve as early warning signals for inflammatory responses and tissue destruction. Additionally, microbial genetic markers are increasingly utilized in diagnostics due to advancements in genomics. This novel testing approach can accurately identify potential pathogens and assess the risk of preterm labor and rupture of membranes by analyzing their metabolic activity (137, 141).

### 6.2 Therapeutic strategies

**Figure 4:**
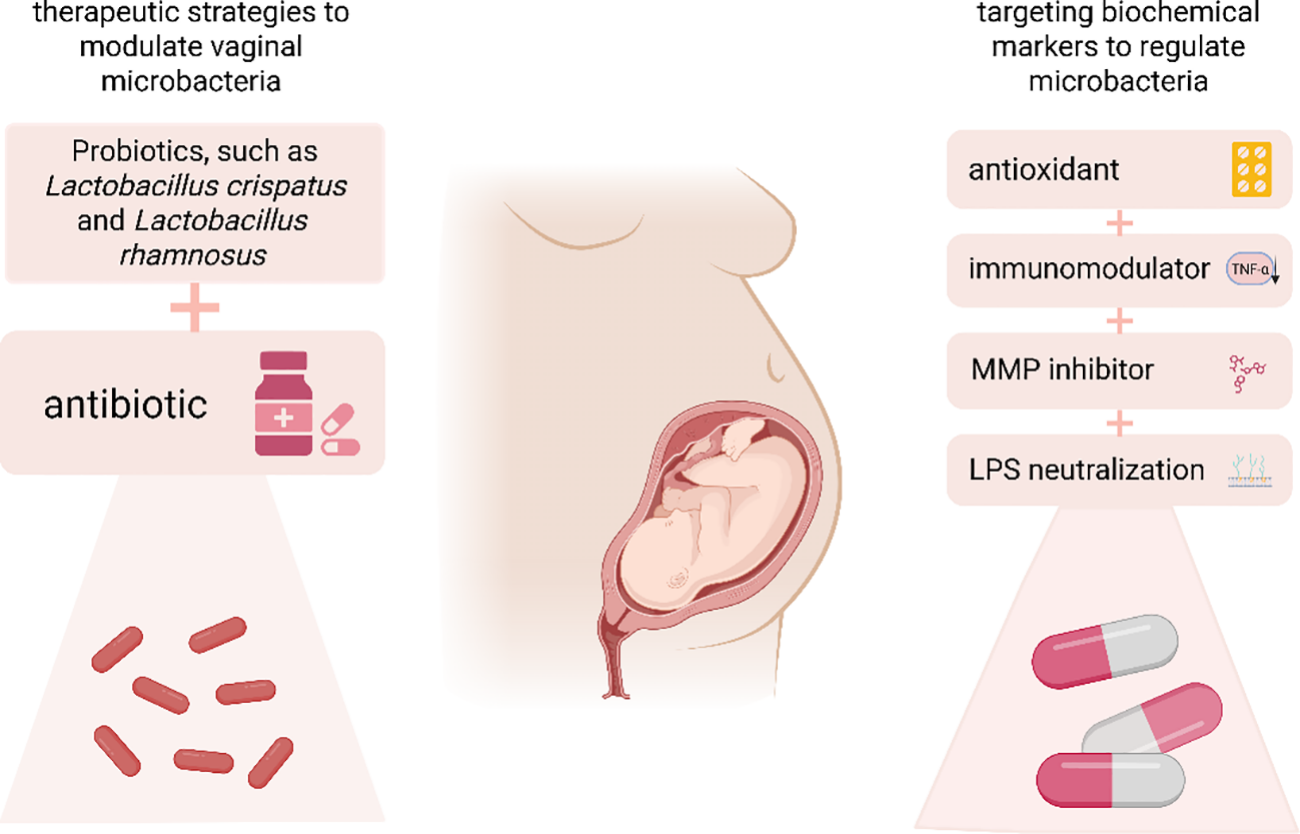
This study presents therapeutic strategies aimed at modulating vaginal microbiota through the use of probiotics, such as *Lactobacillus crispatus* and *Lactobacillus rhamnosus*, in conjunction with antibiotics. Additionally, it explores the targeting of biochemical markers using antioxidants, immunomodulators, MMP inhibitors, and lipopolysaccharides to further influence the vaginal microbiota. MMP, Matrix Metalloproteinase.

The present study proposes a therapeutic approach involving the concurrent administration of probiotics (e.g., Lactobacillus and Bifidobacterium) and antibiotics to regulate the vaginal microbiota (**Figure 4**).

#### 6.2.1 Therapeutic strategies to modulate vaginal microbiota to influence biochemical markers

The regulation of vaginal microbiota has garnered significant attention in recent years as a potential therapeutic strategy for addressing premature rupture of membranes (74, 142). The application of probiotics, such as *Lactobacillus crispatus* and *Lactobacillus rhamnosus*, has been shown to restore *Lactobacillus* dominance in the vagina, thereby mitigating the inflammatory response and the overexpression of MMPs. Clinical studies indicate that regular probiotic use is associated with decreased levels of pro-inflammatory biochemical markers, reduced colonization by pathogenic bacteria, and a lower risk of preterm labor and PROM (143). Furthermore, antibiotic therapy may be employed to regulate vaginal microbiota, particularly in patients diagnosed with bacterial vaginosis or aerobic vaginitis (142). However, the use of antibiotics must be approached with caution, as they can disrupt normal microbiota and potentially exacerbate the inflammatory response. Consequently, a combined approach utilizing both probiotics and antibiotics, which targets pathogenic bacteria while promoting the restoration of beneficial microbiota, has emerged as a promising therapeutic strategy. Some studies have demonstrated that this combination therapy yields improved outcomes, with probiotics effectively reducing the expression of pro-inflammatory markers while restoring microbial balance. This dual approach not only addresses infections in the short term but also diminishes the risk of reinfection and inflammatory flare-ups by maintaining long-term microbiota stability (144). Additionally, personalized probiotic therapies tailored to individual microbiota differences are increasingly being investigated and may serve as a crucial tool in the prevention and treatment of preterm rupture in the future. However, its clinical application still faces multiple challenges. The primary challenge lies in the precise identification of key functional strains. Vaginal microbiota exhibit significant variability among individuals (e.g., classification based on community state type (CST)), and it remains unclear which combination of lactobacilli (e.g., a single strain of L. crispatus or a mixed population of L. gasseri and L. crispatus) is most effective in treating specific types of dysbiosis. Furthermore, the colonization, persistence, and stability of exogenous probiotics are inadequate, with vaginal colonization rates typically falling below 30%. This is partly due to competitive exclusion effects from the native microbiota (e.g., L. iners inhibits the proliferation of exogenous strains through metabolic products). The high heterogeneity of host responses further complicates matters—individual genetic backgrounds, immune states, and local microenvironmental factors (such as pH fluctuations and differences in cervical mucus composition) can significantly influence the metabolic activity and immunomodulatory efficacy of probiotics. The anti-inflammatory effects of the same strain can vary by up to fivefold across different hosts, further hindering the precision of interventions. These challenges collectively impede the standardized application and predictability of the efficacy of probiotic therapy in clinical settings.

#### 6.2.2 Therapeutic strategies for targeting biochemical markers to regulate microbiota

The management of premature rupture of membranes heavily relies on the regulation of biochemical markers. This can be achieved by either modulating the inflammatory response or suppressing the overactivity of MMPs, the degradation of fetal membranes can be mitigated, thereby delaying or preventing premature rupture. Recent studies indicate that antioxidants can effectively lower levels of oxidative stress and diminish ROS-induced damage to fetal membranes. For instance, vitamins C and E, as antioxidants, can inhibit ROS generation and protect fetal membrane cells from oxidative stress-related damage (145, 146). In the realm of anti-inflammatory therapy, certain immunomodulatory drugs, such as TNF-α inhibitors, have demonstrated promising therapeutic effects (147). These agents reduce the release of pro-inflammatory factors, thereby interrupting the inflammatory cascade and decreasing MMP activity. Furthermore, biological drugs like MMP inhibitors, which specifically target MMP activity to reduce fetal membrane degradation, are currently under preclinical investigation (71, 121). Additionally, probiotic treatments that modulate microbiota can protect fetal membranes by diminishing the metabolites of pathogenic bacteria, such as LPS, and significantly reducing the aberrant expression of biochemical markers[ (73, 123). By integrating multiple treatment modalities, including probiotics, anti-inflammatory agents, and antioxidants, future therapeutic strategies can become more comprehensive and individualized, providing a variety of options for patients at high risk of premature rupture of membranes and preterm labor.

#### 6.2.3 Emerging microbiota-targeted therapies

In recent years, significant breakthroughs have been achieved in the prevention and treatment of vaginal microecological imbalances and premature rupture of membranes (PROM). The focus has centered on three main areas: probiotic intervention, vaginal microbiota transplantation (VMT), and biofilm-targeted therapy. A key advancement in probiotics has been the selection of specific strains (148). A study by Short et al. involving HIV-positive pregnant women demonstrated that the presence of Lactobacillus in the gut significantly reduces the expression of pro-inflammatory factors (IL-6 and TNF-α) and matrix metalloproteinases (MMP-9), thereby lowering the risk of preterm birth (149). Large-scale clinical trials have shown that probiotics can decrease the risk of recurrent preterm PROM in high-risk pregnant women by 30% to 40%, while also extending the average gestational age at pregnancy termination by two to three weeks (150). Further randomized clinical trials have confirmed that using probiotic preparations containing L. crispatus during mid-pregnancy can reduce the risk of recurrent preterm PROM (P = 0.006) (151). Prebiotics, such as oligosaccharides, enhance the acid barrier by promoting the growth of lactobacilli, which indirectly inhibits biofilm formation (152). For high-risk groups, such as those with a history of preterm birth or bacterial vaginosis (BV), probiotic treatment with L. rhamnosus GR-1 can normalize vaginal microbiota in mid-pregnancy and reduce GBS colonization by 40% (153). Treatment plans should be personalized and optimized based on microbial community genotyping (e.g., intervention for L. iners-dominant differentiation) and metabolomics (e.g., short-chain fatty acid levels).

Vaginal microbiota transplantation (VMT) is an emerging therapy that rapidly restores the microecological balance centered on lactobacilli by transplanting healthy donor microbiota (154). The first human clinical trial confirmed its effectiveness against recurrent bacterial vaginosis (BV). However, strict donor screening is essential to prevent the transmission of pathogenic bacteria, such as ensuring that the abundance of L. crispatus exceeds 70% (155). Current challenges include verifying long-term safety, standardizing transplantation methods (e.g., lyophilized preparations versus fresh samples), and establishing ethical guidelines.Biomembrane-targeted therapy represents an innovative solution to drug resistance. Enzymes such as lysozyme and DNase can degrade the extracellular polymers present in biomembranes (156). New anti-biofilm agents, including phages, can specifically lyse pathogenic bacteria without harming commensal bacteria (157). Furthermore, targeted delivery systems, such as pH-responsive nanogels, have been shown to enhance the penetration efficiency of drugs into the deeper layers of the vaginal epithelium (158, 159).

In summary, contemporary treatment strategies are increasingly integrating multimodal approaches: probiotics provide fundamental microecological regulation, VMT facilitates rapid reconstruction, and biofilm-targeted therapies overcome the challenges of drug resistance. In the future, it will be crucial to incorporate multi-omics technologies (such as spatial transcriptomics) to analyze the dynamic interactions between the microbiota and the host, promote individualized treatment plans and phased clinical trials, and fundamentally revolutionize the prevention and treatment system for PROM.

### 6.3 Preventive strategies and standardised assessment of vaginal health

#### 6.3.1 Microbiota state typing (CST)-guided prevention

Different CST types necessitate strain-specific probiotic regimens. The optimal condition is CST-I (Lactobacillus crispatus-dominant), which can be sustained through the oral intake of Lactobacillus crispatus strains, such as L. crispatus DSM31983. This regimen maintains a low pH (approximately 4.0), inhibiting the adherence of pathogens and consequently reducing the risk of preterm birth (43). In the case of CST-III type, characterized by Lactobacillus acidophilus dominance, vaginal suppositories containing Lactobacillus rhamnosus GR-1 and Lactobacillus gasseri (10^9 CFU/day for 10 days) have been shown to significantly extend the gestational latency period in patients experiencing preterm PROM. This effect is attributed to the enhancement of local immune defensin HBD-2 secretion and the regulation of the placental TLR signaling pathway. For CST-IV/V type, which consists of a mixed dominance of anaerobic bacteria, a biofilm-targeted therapy is necessary: following the degradation of the biofilm matrix by lysozyme, Lactobacillus rhamnosus CA15 (10^10 CFU/ml) or PB01 strain (>10^8 CFU/ml) is administered sequentially for 10 days, resulting in an increase in the proportion of lactobacilli to 62 ± 8% while concurrently reducing pro-inflammatory factors IL-6/TNF-α (160). For women with a history of preterm birth or concurrent PROM, extending the probiotic treatment duration to 28 days significantly decreases the incidence of PROM (relative risk (RR) = 0.42, p < 0.001) (133, 160). It is crucial to acknowledge that the protective effects of orally administered probiotics on asymptomatic women remain contentious. Additionally, the probiotic dosage should be adjusted based on dynamic monitoring of vaginal pH (161, 162). There is a lack of consensus regarding the clinical significance of CST-III microbiota. Some researchers hypothesize that this condition may evolve into a mixed type (CST-IV). To achieve a more refined classification, it is essential to incorporate host immune markers, such as HBD-2 levels. Furthermore, while vaginal microbiota transplantation (VMT) has been shown to reduce the risk of PROM by 40% in animal models, the long-term safety of this procedure and its standardized methodology require further validation.

#### 6.3.2 Dynamic monitoring of vaginal pH

Vaginal pH dynamic monitoring serves as a crucial tool for assessing the microecological balance of the female reproductive tract, with its fluctuations closely associated with pregnancy outcomes and the risk of PROM. A normal vaginal environment depends on a microecological balance predominantly maintained by lactobacilli, which ensure an acidic pH (≤4.5) that inhibits pathogen colonization through lactic acid metabolism. A sustained pH exceeding 4.7 indicates a microecological imbalance, such as bacterial vaginosis (BV), which is significantly correlated with an increased risk of PROM (163). When the pH level rises above 4.7, the population of lactobacilli diminishes, facilitating the proliferation of anaerobic bacteria, such as Gardnerella, and activating the TLR pathway. This activation leads to the release of pro-inflammatory factors, including IL-6 and TNF-α (133). These inflammatory responses induce oxidative stress (elevated reactive oxygen species, ROS), activating matrix metalloproteinase MMP-9, which degrades collagen in the amniotic membrane, thereby increasing its fragility. In early pregnancy, a pH level greater than 4.7, coupled with reduced levels of defensin HBD-2, heightens the risk of PROM by 2.3 times. Fluctuations in pH levels exceeding 0.5 units per week indicate a shift in the microbiota toward a high-risk type (CST-IV), necessitating a more refined classification based on immune markers (163). Smart tampon sensors facilitate real-time monitoring of vaginal pH at home, addressing the limitations of traditional single-visit clinic testing. Dynamic monitoring of vaginal pH is a key indicator for predicting PROM. By identifying early dysbiosis and guiding targeted probiotic interventions alongside inflammation marker analysis, these sensors provide precise strategies for improving pregnancy outcomes. Moving forward, it is essential to promote standardized home monitoring technologies and integrated prediction models that consider both microbiome and immune factors.

## 7 Challenges and prospects

### 7.1 Challenges

While the current study elucidates the mechanisms of interaction between vaginal microbiota and biochemical markers in PROM, several challenges remain in clinical application and research practice. First, the reproducibility and stability of study results require further attention. The high variability of vaginal microbiota among individuals, coupled with the complex array of factors influencing microbiota—such as genetic background, environmental influences, and lifestyle habits—often complicates the standardization of results across different studies. Thus, validating these findings in larger clinical trials and ensuring the reliability of the results represent significant challenges at present. Second, the difficulties associated with clinical translation must not be overlooked. The assessment of the safety and efficacy of treatments necessitates additional clinical trial data. For instance, although probiotic therapies have demonstrated potential in regulating vaginal microbiota, their long-term safety and optimal use in conjunction with antibiotics and anti-inflammatory drugs warrant thorough investigation (16). In addition, therapies targeting biochemical markers, such as MMP inhibitors, require rigorous clinical validation to confirm their efficacy in patients at high risk of premature rupture.

### 7.2 Prospects

In the future, advancements in multi-omics research techniques (e.g., metabolomics, microbiomics, transcriptomics) will enable a more precise and in-depth study of the interactions between vaginal microbiota and biochemical markers. By integrating multi-omics approaches, we can achieve a comprehensive understanding of the metabolic functions of vaginal microbiota, microbe-host interactions, and the dynamics of biochemical markers. This comprehensive approach will enable the identification of novel pathological mechanisms linked to the premature rupture of membranes, thus establishing a basis for tailored diagnostic and therapeutic strategies. In terms of clinical implications, an improved comprehension of how vaginal microbiota interacts with biochemical markers could strengthen the application of personalized medicine in preventing and managing premature rupture of membranes. Future treatment trends may include combined probiotic therapy, marker-based targeted therapies, and the concurrent use of antioxidants and anti-inflammatory drugs for individuals at high risk of premature rupture. Furthermore, the development of convenient and efficient tools for testing vaginal microbiota and biochemical markers will significantly improve the accuracy of clinical diagnoses and the effectiveness of early interventions.

## 8 Conclusions

Research has highlighted the significant interaction between vaginal microbiota and biochemical markers in instances of premature rupture of membranes. Dysbiosis within the microbiota is strongly associated with changes in the expression of pro-inflammatory cytokines, matrix metalloproteinases (MMPs), and various other biochemical markers.These elements interact through inflammatory responses and oxidative stress pathways, ultimately influencing the stability of fetal membranes. Based on these findings, a comprehensive evaluation of vaginal microbiota along with biochemical indicators presents new strategies for the early identification and tailored management of premature rupture of membranes.Despite notable advancements in research, challenges persist, particularly regarding the reproducibility of results and the clinical applicability of findings. Looking ahead, the advancement of multi-omics technology promises to enhance our understanding of vaginal microbiota and biochemical markers, thereby providing more effective tools and strategies for the clinical management of preterm birth. Future research and clinical practice should prioritize improving the accuracy of diagnostic tools and tailoring treatments to enhance pregnancy outcomes and mitigate the effects of preterm birth on maternal and infant health.
